# Integrating and Simplifying Evidence to Optimize Cardiorenal Guideline-Directed Therapies

**DOI:** 10.3390/jcm14165883

**Published:** 2025-08-20

**Authors:** Harleen Singh, Carrie Puckett, Yennie Q. Lucas

**Affiliations:** 1 School of Pharmacy, The University of Texas at El Paso, 500 W. University Avenue, El Paso, TX 79968, USA; 2 Department of Cardiology, Veterans Health Administration, 3710 SW US Veterans Hospital Road, P3Cards, Portland, OR 97239, USA; carrie.puckett@va.gov; 3 Department of Clinical Pharmacy Services, Kaiser Permanente Northwest, 5717 NE 138th Avenue, Portland, OR 97230, USA; yennie.q.lucas@kp.org

**Keywords:** chronic kidney disease, heart failure, cardiorenal outcomes, guideline-directed medical

## Abstract

Chronic kidney disease (CKD) prevalence is rising worldwide and is projected to become the fifth leading cause of death globally by 2040. The high proportion of undiagnosed early-staged CKD and delayed diagnosis is of significant concern. The access to diagnosis and treatment is also limited in low-resource settings. The majority of individuals with kidney disease succumb to cardiovascular disease complications. Furthermore, heart failure and CKD are closely interconnected, with each condition significantly increasing the risk of developing the other. They share common risk factors, such as high blood pressure and diabetes, and their coexistence worsens prognosis and raises mortality rates. The bidirectional relationship between the heart and kidneys becomes even more complex and challenging in the context of cardiorenal syndrome. Emerging medications, such as sodium–glucose cotransporter 2 inhibitors and mineralocorticoid receptor antagonists, have shown remarkable efficacy in slowing the progression of kidney disease, surpassing the benefits of traditional treatments. This article summarizes the evidence on the early detection of CKD and real-world opportunities to slow the progression of CKD by optimizing cardiorenal guideline-directed medical therapy.

## 1. Introduction

The global prevalence of chronic kidney disease (CKD) is steadily increasing, currently affecting an estimated 850 million individuals worldwide. If left untreated, CKD is progressive and may ultimately lead to kidney failure requiring kidney replacement therapy (KRT). Despite its growing burden, CKD remains significantly underdiagnosed and under-treated, particularly in its early stages. Current epidemiological projections suggest that by 2040, CKD will become the fifth leading cause of mortality globally [1,2]. CKD imposes a substantial economic burden on both individuals and healthcare systems. In high-income countries, KRT—including hemodialysis and peritoneal dialysis—accounts for approximately 2–3% of total annual healthcare expenditures [3]. By 2032, the global cost of KRT across eight major economies (the United States, Brazil, the United Kingdom, Spain, Germany, the Netherlands, China and Australia) is projected to approach USD 186 billion [4].

CKD and cardiovascular (CV) disease share numerous pathophysiological risk factors, including hypertension (HTN) and diabetes mellitus (DM). Their coexistence substantially worsens clinical outcomes and increases mortality. The interplay between renal and cardiac dysfunction is further complicated in the context of cardiorenal syndrome, a condition characterized by bidirectional organ impairment that presents complex diagnostic and therapeutic challenges. Beyond clinical complications, individuals living with CKD often endure considerable morbidity with an impaired quality of life [1,5]. Early detection is a key strategy to prevent kidney disease, its progression and related complications, but numerous studies show that awareness of kidney disease at the population level is low. Therefore, increasing knowledge and implementing sustainable solutions for early detection of kidney disease are public health priorities. Economic and epidemiological data underscore why kidney disease should be placed on the global public health agenda—kidney disease prevalence is increasing globally, and it is now the seventh leading risk factor for mortality worldwide. Moreover, demographic trends, the obesity epidemic and the sequelae of climate change are all likely to increase kidney disease prevalence further, with serious implications for survival, quality of life and health care spending worldwide. Importantly, the burden of kidney disease is highest among historically disadvantaged populations that often have limited access to optimal kidney disease therapies, which greatly contributes to current socioeconomic disparities in health outcomes. The joint statement from the International Society of Nephrology, European Renal Association and American Society of Nephrology, supported by three other regional nephrology societies, advocates for the inclusion of kidney disease in the current WHO statement on major non-communicable disease drivers of premature mortality [1]. The disease is associated with physical limitations, psychosocial distress, cognitive impairment, employment challenges, social isolation and increased risk of premature death [6].

Recent therapeutic advances—particularly the emergence of sodium–glucose co-transporter 2 inhibitors (SGLT2is) and nonsteroidal mineralocorticoid receptor antagonists (ns-MRA)—have demonstrated significant efficacy in slowing CKD progression and improving cardiorenal outcomes, surpassing the benefits observed with conventional therapies. Based on evolving evidence, the 2024 Kidney Disease: Improving Global Out-comes (KDIGO) guidelines for CKD highlight early detection, lifestyle recommendations and a multidisciplinary approach to care [7].

This article reviews current evidence on the early detection, diagnosis and treatment of CKD and highlights real-world opportunities to prevent disease progression through the implementation of guideline-directed medical therapy (GDMT) targeting both renal and CV systems, thereby improving outcomes.

## 2. Catching Chronic Kidney Disease Early: Why It Matters

In the primary care setting, CKD is often first identified—but frequently overlooked. As the main point of contact for routine annual evaluations, primary care providers (PCPs) are typically responsible for monitoring blood pressure, assessing urinary albumin excretion and estimating glomerular filtration rate (eGFR). Abnormal findings in any of these areas should ideally lead to early recognition and diagnosis of CKD. However, current evidence suggests that CKD is frequently underdiagnosed or diagnosed late in the primary care setting [8].

Several barriers contribute to this diagnostic delay. A major factor is limited awareness or understanding of CKD among PCPs, which also affects patient awareness. Additionally, clinical guidelines for CKD are often perceived as complex, comprising multiple recommendations with varying levels of supporting evidence. This complexity can hinder effective implementation in routine practice [9]. Furthermore, differentiating between acute and chronic changes in GFR requires additional diagnostic steps such as quantifying proteinuria, which is critical for staging of CKD.

The multinational Patient Management and Clinical Outcomes Associated with a Recorded Diagnosis of Stage 3 Chronic Kidney Disease (REVEAL CKD) observational study was launched to quantify the prevalence of previously undiagnosed Stage 3 chronic kidney disease using real-world data from multiple countries [10]. The study explored the effects of diagnostic delays and evaluated how healthcare providers manage CKD after the confirmed diagnosis. A key finding of the REVEAL-CKD study was that a significant number of patients with confirmed Stage G3 CKD remained undiagnosed by their general practitioners. Strikingly, the prevalence of undiagnosed CKD was nearly the same in patients with recognized risk factors—such as DM, heart failure(HF), or HTN—and those without these conditions. One possible explanation was that clinicians may hesitate to formally code CKD in patients already receiving antihypertensive treatments, particularly renin-angiotensin system inhibitors (RASis), under the assumption that these therapies also address underlying kidney impairment [10]. Additionally, patients who eventually received a CKD diagnosis typically experienced notable decline in kidney function over time, which likely triggers diagnostic coding. In contrast, those with stable eGFR values are less frequently diagnosed, even when they meet clinical criteria for CKD.

In an interview summary, Tangri and De Nicola highlighted that CKD is often asymptomatic in its early stages, with symptoms typically appearing only after more than 70% of kidney function has been lost. Without timely diagnosis, 40% of patients with Stage 3 CKD progress to Stage 4 or 5 within a year, and about 60% eventually require dialysis or a kidney transplant—at which point treatment options become more limited. Early detection, particularly in stages G1 to G2 using albuminuria as a marker, is essential for initiating timely interventions and preventing progression to end-stage kidney disease (ESKD). In contrast, diagnosis at Stage G4 offers fewer opportunities to alter the disease course and may only delay the need for KRT [11,12]. Additional analysis of the REVEAL-CKD study further showed that a documented CKD diagnosis is linked to improved disease management, better monitoring and a slower decline in kidney function [12]. For a busy PCP, integrating the nuanced evaluation required for early CKD diagnosis and management can be challenging. CKD is frequently perceived as a secondary complication of conditions such as DM and HTN, rather than as an independent disease entity. This perspective may contribute to the underuse of evidence-based therapies that can slow CKD progression [12]. Addressing these gaps through improved education, streamlined guidelines and decision-support tools may enhance early detection and management of CKD in primary care settings.

## 3. GFR and UACR: The Dynamic Duo for Detecting, Staging and Tracking CKD

### 3.1. GFR

Glomerular filtration rate (GFR) is a central parameter in the assessment of kidney function. Multiple methods are available to evaluate GFR, with estimated GFR based on serum creatinine (eGFRcr) being the commonly used initial test. This preference is largely due to its broad availability, standardized laboratory methods and endorsement by clinical guidelines.

**Figure 1:**
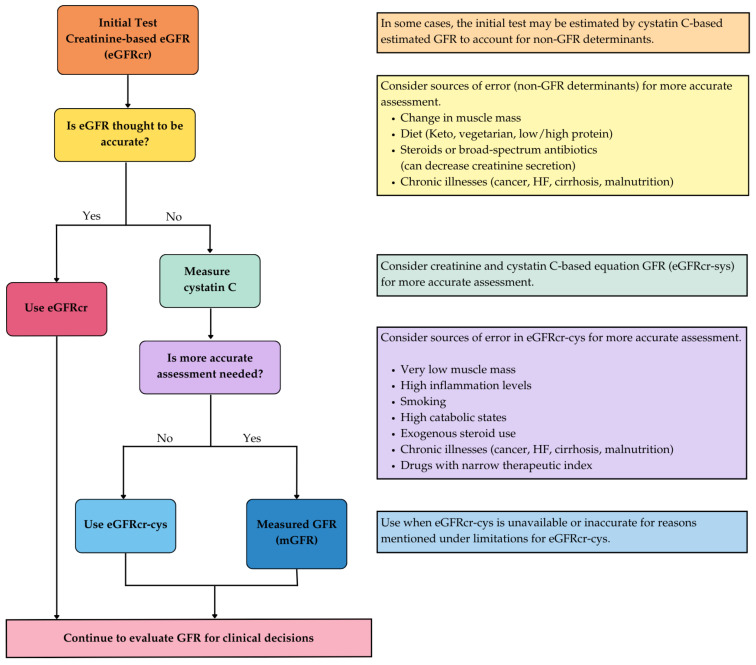
Methods for assessing glomerular filtration rate (GFR). Adapted with permission from [7].

The KDIGO guidelines recommend using eGFRcr as the first-line assessment. However, in situations where the accuracy of creatinine-based estimates may be compromised—such as in individuals with extremes of muscle mass, malnutrition or other con-founding factors—it is advisable to perform a confirmatory assessment using serum cystatin C. When cystatin C is measured, it should be incorporated into a validated estimating equation to provide a cystatin C–based eGFR (eGFRcr-cys) [7]. An algorithm to assess GFR is shown in Figure 1.

While these estimating equations are widely validated and are cost-effective for routine clinical use, they are not without limitations. Interpretation must be cautious in non-steady-state conditions, such as acute kidney injury (AKI) or rapidly fluctuating renal function, where estimations may not accurately reflect true GFR.

For select clinical scenarios requiring precise quantification of renal function, direct measurement of GFR using exogenous filtration markers (e.g., inulin, iohexol or iothala-mate clearance) may be warranted. Although considered the gold standard, measured GFR methods are resource-intensive, often costly and impractical for routine or population-level screening. When using eGFRcr, it should not be interpreted as a precise point estimate but rather as a range within which the true GFR likely resides. In contrast, measured GFR—when performed using exogenous filtration markers—demonstrates significantly less variability and a narrower confidence interval across repeated assessments, making it a more accurate representation of true kidney function in select clinical contexts but not for routine use [7,13]. In the context of CKD monitoring, it is essential to distinguish between expected biological variability and clinically meaningful changes in renal function [14].

### 3.2. Albumin-to-Creatinine Ratio (uACR)

A state-of-the-art review published in the Journal of the American College of Cardiology highlighted the prognostic utility of albuminuria in cardiovascular outcomes. Specifically, albuminuria is a robust predictor of incident HF and is also associated with the progression of established HF. Notably, its predictive capability surpasses that of eGFRcr. Furthermore, the regression of albuminuria has been linked with a reduction in the risk of HF events [15].

Assessment of albuminuria is most effectively performed via the urine albumin-to-creatinine ratio (uACR) in a random spot urine sample. Alternative methods, such as 24 h urine collections, are logistically burdensome and offer limited additional predictive value. Albuminuria is categorized based on uACR (Figure 1). It is important to account for biological variability in uACR measurements, which may exceed 20%. Therefore, confirmation of abnormal albuminuria requires 2–3 elevated readings obtained over a period of 3 to 6 months [7]. Early changes in the kidney may be detected by an increase in albuminuria before changes in eGFR are evident. Therefore, albuminuria serves as a marker of early progressive structural kidney damage in patients with Type 2 diabetes mellitus (T2DM). Albuminuria also contributes to inflammation and fibrosis in CKD [16,17].

**Figure 2:**
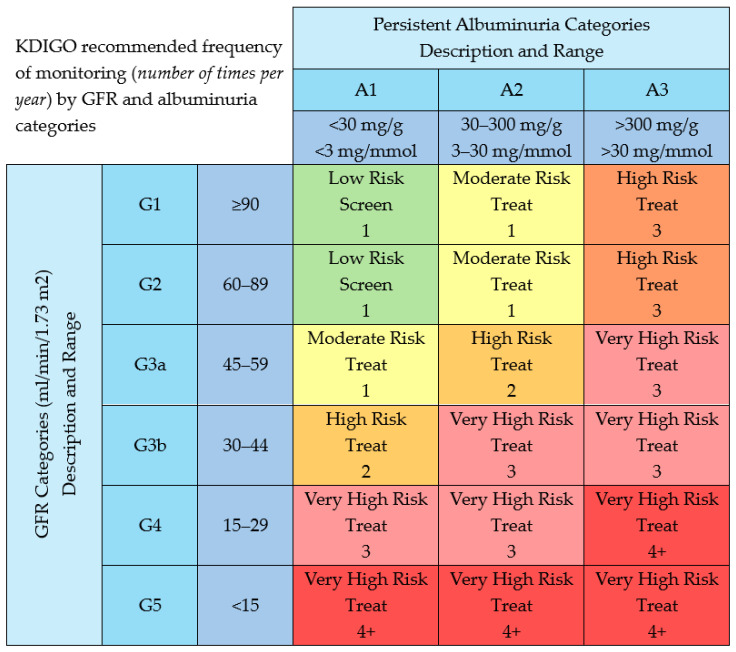
Staging of chronic kidney disease (CKD). The numbers in the boxes are a guide to the frequency of monitoring (number of times per year). Adapted with permission from reference [7].

In the context of CKD, uACR functions as an independent predictor of CV mortality, outperforming eGFR in this regard [18]. The relationship between uACR and CV mortality is continuous and graded, with increased risk observed even within values traditionally considered normal. For instance, uACR levels exceeding 10 mg/g have been associated with elevated CV mortality risk, and levels up to 29 mg/g, although below the conventional threshold, still carry prognostic significance [18]. Monitoring frequency for uACR should be tailored according to the individual’s risk of CKD progression and associated complications. Current recommendations suggest repeat screening at intervals ranging from annually to every 3–4 months, depending on clinical context and underlying risk stratification [7]. The primary goal in managing albuminuria in CKD is to achieve a 30% reduction in urinary uACR. Reaching this target has been linked to slower progression of CKD. However, a major clinical challenge remains: 40–50% of patients do not achieve this reduction, even when treated with RASis [19]. Therefore, the KDIGO framework for categorizing renal risk—based on both eGFR and uACR—provides essential guidance for identifying patients at increased risk of kidney disease progression and associated CV complications. Figure 2 shows when to treat and monitor frequency of CKD based on staging.

Furthermore, KDIGO recommends the concurrent assessment of eGFR and uACR to more accurately evaluate renal function. The uACR is particularly critical, as it has been shown to independently predict adverse outcomes in patients with HF, regardless of baseline eGFR [15]. Notably, individuals with elevated uACR and preserved eGFR still exhibit poorer clinical outcomes. Individuals with CKD and normoalbuminuria (<30 mg/g) are also at an increased risk of CKD progression, with this risk rising linearly as albuminuria levels increase [19]. Albuminuria serves as a surrogate marker of podocyte injury and reflects underlying endothelial dysfunction, underscoring its prognostic significance [20]. Real-world screening for albuminuria is still suboptimal, given that elevated levels can increase the CV risk and renal morbidity and mortality. Prompt, effective treatments that target albuminuria are therefore crucial to improve outcomes for individuals with current or future CV diseases.

## 4. Renal Troubles: Sorting out Acute Kidney Injury (AKI) from Worsening Renal Function (WRF)

In clinical practice, the terms AKI and WRF are at times used interchangeably in patients presenting with acute decompensated heart failure (ADHF). There are varying definitions of AKI, with the KDIGO guidelines defining AKI or kidney dysfunction based on changes in serum creatinine (SCr), ranging from an increase of 0.3 mg/dL to SCr increase by at least 1.5 times of baseline and/or decline in urine volume (0.5 mL/kg/h for 6 h duration) [21]. Many pharmacotherapies used in HF management can impact renal function via various modalities.

In normal physiology, homeostasis between the kidneys and heart is well maintained. However, in ADHF, this balance can be disrupted. Decongestion may lead to a temporary reduction in renal artery blood flow and impaired renal venous return—changes that are often reversible. In contrast, AKI from other causes, such as sepsis, nephrotoxic drugs or prolonged severe hemodynamic compromise, may be irreversible and is linked to poor prognosis [22]. It is difficult to predict whether the rise in SCr following the initiation of decongestive therapy will be transient or continue to worsen, potentially leading to KRT. It also highlights the dynamic and complex heart kidney interplay in cardiorenal syndrome, which is expansive and not within scope for this review.

Acute declines in eGFR caused by medications that affect kidney perfusion—such as SGLT2is and RASis—are generally well tolerated. A transient small initial decline in eGFR is due to reduction in intraglomerular pressure, compared to AKI, where a sudden rise in SCr, usually above 30%, can be due to renal insult, requiring further evaluation. The GDMT-related transient changes should not immediately prompt discontinuation of therapies that provide long-term CV and renal benefits. When part of an intentional strategy is to reduce long-term risk, this phenomenon may be more appropriately described as “permissive AKI” or “permissive hypercreatininemia” [23]. It is essential to closely monitor and account for all potential contributors to renal dysfunction and to confirm its transient nature through repeat blood work.

On the contrary, in CKD, decline in eGFR progresses more slowly after introduction of GDMT, and in some cases, eGFR can deteriorate during the first weeks after GDMT initiation. However, interpreting these early changes in renal function remains challenging due to the absence of standardized protocols for timing renal function assessment in response to treatment. Furthermore, follow-up durations in both clinical practice and trials are often too short to capture meaningful long-term renal outcomes, undermining the interpretability of short-term renal changes as reliable surrogates for CKD progression. This challenge is further compounded by the significant heterogeneity observed in clinical trials involving patients with chronic heart failure. Variability in inclusion criteria and inconsistent definitions of renal endpoints—particularly the use of composite outcomes that blend hard renal events with surrogate markers—complicate interpretation and hinder meaningful comparisons across studies. Nevertheless, GDMT should be introduced slowly and with caution in CKD, followed with diligent monitoring.

**Figure 3:**
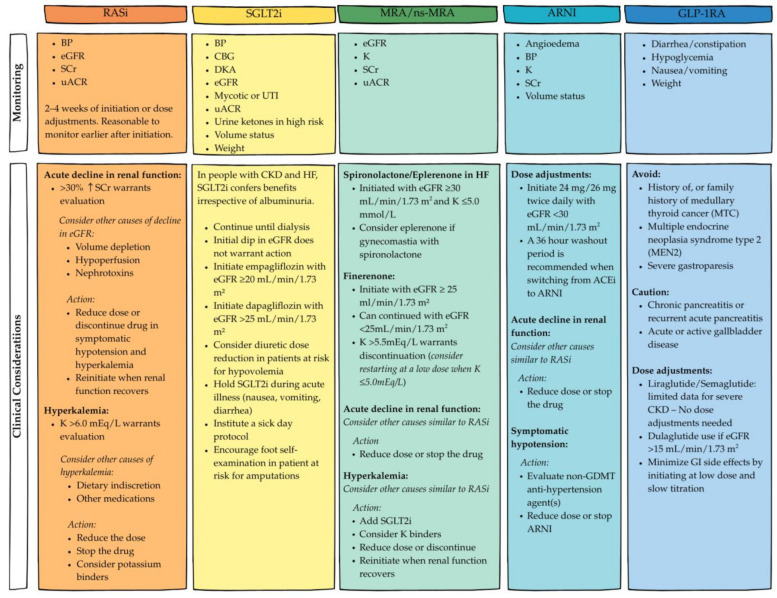
Clinical considerations and key monitoring parameters for GDMT for cardiorenal care. Adapted from references [7,27]. Abbreviations: BP = blood pressure, K = potassium, eGFR = estimated glomerular filtration rate, SCr = serum creatinine, uACR = urine albumin-to-creatinine ratio, CBG = capillary blood glucose, CKD = chronic kidney disease, DKA = diabetic ketoacidosis, SGLT2i = sodium–glucose co-transporter 2, GLP-1RA = glucagon-like peptide-1 receptor agonist, RASi = renin-angiotensin system inhibitor, UTI = urinary tract infection.

As mentioned above, careful interpretation of eGFR decline associated with each GDMT is crucial during therapy titration or discontinuation, which is shown in Figure 3. The Angiotensin–Neprilysin Inhibition in Heart Failure with Preserved Ejection Fraction (PARAGON-HF) trial [24] compared sacubitril/valsartan (S/V) to valsartan (an active comparator); an initial decline in eGFR was observed in both treatment arms, consistent with the known hemodynamic effects of angiotensin receptor blockers (ARBs). However, S/V demonstrated a slower rate of eGFR decline over time, with a clear and sustained separation of eGFR trajectories favoring S/V throughout the follow-up period. In contrast, the Dapagliflozin in Heart Failure with Mildly Reduced or Preserved Ejection Fraction (DELIVER) trial [25] evaluated dapagliflozin versus a placebo, showing a different pattern. Dapagliflozin was associated with an acute decline in eGFR of approximately 3–4 mL/min/1.73 m^2^, followed by stabilization. The eGFR curves for dapagliflozin and the placebo crossed after approximately 24 months, reflecting a well-recognized eGFR slope pattern associated with SGLT2is. This acute dip followed by long-term preservation of kidney function is considered a class effect of SGLT2is and should be interpreted as a benign, expected phenomenon. Similarly, in the Finerenone in Patients with Heart Failure with Mildly Reduced or Preserved Ejection Fraction (FINEARTS-HF) trial [26] evaluating finerenone versus a placebo, an initial eGFR reduction was noted with finerenone; however, the long-term eGFR slopes were nearly parallel between the two groups, indicating that while an acute hemodynamic effect is present, it does not appear to accelerate kidney function decline over time. Understanding these distinct patterns is essential for appropriate interpretation of eGFR changes following initiation of therapy. These changes should be assessed in light of each agent’s mechanism of action and expected physiological impact.

Regarding the prognostic implications of these acute eGFR changes, a pre-specified secondary analysis from the DELIVER trial provides useful insights. Patients who experienced an acute eGFR decline >10%—referred to as “dippers”—while on dapagliflozin did not exhibit an increased incidence of the primary CV outcome. In contrast, a similar decline in eGFR among patients receiving a placebo was associated with worse outcomes, suggesting that the eGFR reduction observed with dapagliflozin is not detrimental and is likely hemodynamically mediated rather than indicative of structural kidney injury [28]. Further support for the reversibility of these changes comes from the study of sotagliflozin in patients with DM and CKD [29]. In this trial, withdrawal of the drug resulted in a recovery of eGFR, reinforcing the hemodynamic nature of the acute eGFR reduction. A similar recovery pattern was observed with empagliflozin upon withdrawal in the EMPEROR program, which included patients with both HF with preserved ejection fraction (HFpEF) and HF with reduced ejection fraction (HFrEF) [30].

In summary, a transient rise in serum creatinine is usually hemodynamic, is not intrinsic renal injury and should be assessed in the context of HF status and GDMT effects. It should not prompt stopping GDMT or decongestive therapy.

## 5. The GDMT Adoption Dilemma in HF with CKD

Renal impairment is a key factor contributing to the underutilization and suboptimal dosing of GDMT in HF. Although strong evidence supports the early initiation of GDMT to reduce mortality, prevent readmissions and enhance quality of life in HF patients, real-world data tell a different story. Findings from the Change the Management of Patients with Heart Failure (CHAMP-HF) registry [31] and the European Society of Cardiology Heart Failure Long-Term Registry (ESC-HF-LT) [32] show that a substantial proportion of outpatients are not prescribed the recommended therapies, and only a small fraction receive target doses. Factors such as advanced age, hypotension, impaired cardiac function, renal dysfunction and recent heart failure hospitalization (HFH) are commonly linked to reduced dosing. Alarmingly, only 1% of eligible patients achieved all guideline-recommended target doses [31].

A recent retrospective analysis of a consecutive cohort of real-world patients with HFrEF revealed a progressive decline in the use of guideline-directed triple therapy (RASi + β-blocker + MRA) as eGFR decreased from hospital discharge to one-year follow-up. Additionally, the proportion of patients reaching target doses of HF medications at both discharge and one year was notably low. Mortality and HFH increased with worsening eGFR at one year. Clinical trial data have demonstrated that these outcomes can improve with the implementation of GDMT. Notably, eGFR at one year remained stable compared to baseline, even among patients with advanced CKD [33]. Recent data from four major trials on HF with mildly reduced ejection fraction (HFmrEF) and HFpEF highlight the significant overlap of various comorbid conditions. The prevalence of DM across these studies ranges from approximately 35% to 50%. Similarly, CKD is present in about 40% to 50% of patients, underscoring its critical role [34]. These findings emphasize that CKD is a major contributor to the cardiorenal-metabolic syndrome frequently observed in patients with HF. Underlying CKD in HF patients significantly increases the risk of hospitalization and mortality. As kidney function declines, the optimization of HF therapies often becomes suboptimal, in part because many GDMTs can have acute adverse effects on renal function. This complexity necessitates balancing the risks and benefits of treatment through a nuanced, individualized approach that takes into account the severity of both CKD and HF, the presence of comorbidities and each patient’s tolerance to medications.

## 6. Therapies Delivering CV Gains in CKD

### 6.1. RAS Inhibitors: Angiotensin-Converting Enzyme Inhibitors (ACEis), Angiotensin II Receptor Blockers (ARBs)

The KDIGO guidelines advocate for the use of ACEis or ARBs as first-line treatment for managing hypertension and proteinuria in patients with CKD and DM. These RASis are recommended at the highest tolerated or permissible doses due to their demonstrated ability to reduce blood pressure and proteinuria, slow the progression of eGFR decline and decrease the risks of kidney failure, CV complications and all-cause mortality [35,36,37,38]. Additionally, RASis have shown beneficial effects in lowering CV morbidity and mortality in individuals with HF [39].

Despite robust clinical evidence supporting their cardiorenal protective properties, real-world data from observational studies indicate that RASis are often prescribed at suboptimal doses in patients with HF and CKD. One major challenge is the limited safety data available for individuals with advanced CKD, particularly those with eGFR below 30 mL/min/1.73 m^2^, as most large-scale CV trials have excluded this population. The question of whether to continue or discontinue RASi therapy once eGFR falls below has been recently explored. Some small observational studies have raised concerns of continuing RASis in advanced CKD [40,41]. However, these findings are limited by small sample sizes and the inherent constraints of observational research.

In a more comprehensive analysis published in 2020, Qiao et al., conducted a retrospective, propensity score-matched cohort study to evaluate the outcomes of continuing versus discontinuing RASis in patients whose kidney function declined to an eGFR below 30 mL/min/1.73 m^2^ [42]. All participants had been on ACEi or ARB therapy and experienced at least one documented drop in eGFR to below this threshold during follow-up. Over a median follow-up of 2.9 years, mortality was higher in the group that discontinued RASi therapy (35%) compared to those who continued (29.4%). Furthermore, those who discontinued therapy exhibited a nearly 40% increased risk of death and major adverse CV events (MACE). While the overall risk of kidney failure requiring KRT did not differ significantly, stratified analysis revealed that patients with diabetes who stopped RASis were at higher risk for KRT than those without diabetes. Based on these findings, the authors concluded that continuing RASis in individuals with declining kidney function may offer CV benefits without substantially increasing the risk of KRT, especially in the absence of T2DM. In addition, several safety outcomes were examined, including the occurrence of hyperkalemia (which was less likely to occur in those who discontinued RASi therapy) and AKI (AKI; with no difference observed by RASi therapy status). Nonetheless, studies, including the one by Qiao et al., are limited by observational design and highlight the ongoing need for randomized clinical trials to inform decision-making in this high-risk population. Given the conflicting risk–benefit profile of RASis in advanced CKD, true equipoise existed. The multicenter randomized controlled trial of angiotensin-converting enzyme inhibitor/angiotensin receptor blocker withdrawal in advanced renal disease (STOP-ACEi) trial was a well-designed, randomized controlled study that aimed to determine whether discontinuing RASis in patients with stage 4–5 CKD could slow disease progression. The trial included a diverse population with various CKD etiologies. Results show no benefit from stopping ACEis or ARBs; in fact, the discontinuation group experienced a 6% higher incidence of KRT and a numerically higher rate of CV events [43]. The decision to continue or stop RASis should be individualized, considering proteinuria, blood pressure control, medication tolerability and CV risk.

### 6.2. Mineralocorticoid Receptor Antagonists (MRAs)

High aldosterone levels from the activation of the RAAS in HF are known to lead to sodium and fluid retention, which ultimately contributes to vasoconstriction, volume expansion and electrolyte imbalances, as well as elevated blood pressure. Beyond its implication in HF, aldosterone is known to impair coronary vascular compliance by increasing oxidative stress with reduction in nitric oxide [44]. Aldosterone ultimately leads to endothelial dysfunction, cardiac remodeling and fibrosis. Not surprisingly, high levels of aldosterone lead to increased risk of CV events and CV mortality [45]. The role of steroidal MRAs and their impact on RAAS system is well established, with impact on clinical practice HF, hypertension and CKD.

Patients with renal dysfunction and HF represent a challenge for clinicians and are often not treated with therapies that could improve outcomes, as previously reviewed. However, the use of steroidal MRA is very low in the context of myocardial infarction (MI) and HF patients with CKD [46,47,48]. This is keeping in mind that MRA randomized controlled trials (RCTs) traditionally have excluded patients with advanced CKD (i.e., sCr > 2.5 mg/dL or eGFR < 20 mL/min/1.73m^2^). The potential WRF and hyperkalemia may lead to fear in clinicians and impact prescription practices.

In a pooled analysis of steroidal MRA RCTs, including Randomized Aldactone Evaluation Study (RALES), Eplerenone in Mild Patients Hospitalization and Survival Study in Heart Failure (EMPHASIS-HF), Treatment of Preserved Cardiac Function Heart Failure with an Aldosterone Antagonist (TOPCAT) and Eplerenone Post-AMI Heart Failure Efficacy and Survival Study (EPHESUS), they demonstrated that MRAs reduced mortality and HF hospitalizations for a wide range of eGFR values. However, they identified that the benefit of MRAs became neutral in patients with eGFR ≤ 30 mL/min/1.73 m^2^ [49]. Additionally, patients with lower eGFR also had more hyperkalemia, as well as worsening kidney function episodes.

As eGFR reduces, the risk of hyperkalemia increases. The use of new novel potassium binders may allow ongoing use of MRAs and other RASis for individuals with HF and CKD. The Study to Assess Efficacy and Safety of Sodium Zirconium Cyclosilicate (SZC) for the Management of High Potassium in Patients with Symptomatic HFrEF Receiving Spironolactone (REALIZE-K) recently showed that SZC led to more patients maintaining normokalemia on goal spironolactone dosages [50]. The impact of potassium binders on clinical outcomes remains unclear. When it comes to behavioral modifications, dietary changes to avoid hyperkalemia should be utilized for all patients on MRAs, especially those with CKD. Concurrent use of SGLT2is with MRAs may reduce the incidence of hyperkalemia [51].

Nonsteroidal MRAs (ns-MRAs) have potential benefits over steroidal MRAs, such as no antiestrogen or antiprogesterone effects, as well as possibly lower risk for hyperkalemia. Unanticipated CV benefits have been found in CKD and DM trials of ns-MRAs. In the cardiovascular and kidney outcomes for finerenone in patients with type 2 diabetes and chronic kidney disease, FIDELITY pooled analysis of two studies (FIDELIO-DKD and FIGARO-DKD) showed that finerenone reduced the risk of clinically significant kidney and CV outcomes across the spectrum of kidney disease [52]. In the Finerenone Trial to Investigate Efficacy and Safety Superior to Placebo in Patients with Heart Failure (FINARTS-HF), those with mildly reduced or preserved ejection fraction had a lower rate of worsening HF events and death from CV causes without any impact on kidney disease progression in a low-risk renal cohort [26]. Despite the preclinical and evolving data with ns-MRAs, real-life use may be limited by the expensive cost compared to generic steroidal MRAs. There are multiple active trials underway to assess steroidal MRAs in HFpEF, as well as their impact in end-stage renal disease. Additionally, the use of na-MRAs is actively being investigated for safety and efficacy in patients with HFrEF.

### 6.3. Angiotensin Receptor–Neprilysin Inhibitor (ARNI)

Clinicians remain cautious in prescribing ARNI for patients with renal dysfunction. However, data from pivotal clinical trials, including the PARADIGM-HF [53] and Prospective Comparison of ARNI with ARB Global Outcomes in Heart Failure with Preserved Ejection Fraction (PARAGON-HF) [23] studies, demonstrated that the efficacy of treatment was consistent irrespective of baseline renal function, with no significant difference observed in treatment effects among patients with eGFR above or below 60 mL/min/1.73 m^2^. Similar results were observed in T2DM subgroups, despite a higher risk of renal decline in DM. Thus, in both HFrEF and HFpEF, the benefits of ARNI in reducing HFH and CV mortality appear to be maintained regardless of the presence of CKD or diabetes.

In a pooled analysis of the PARADIGM-HF and PARAGON-HF trials, treatment with ARNI was associated with a significantly lower incidence of composite renal out-comes [54]. These outcomes included a ≥50% decline in eGFR from baseline, progression to end-stage renal disease, or death attributable to renal causes. Renal outcomes were observed in 1.1% of patients treated with S/V, compared to 1.9% in those receiving either valsartan or enalapril (hazard ratio [HR]: 0.56; 95% CI: 0.42–0.75; *p* < 0.001). The average annual decline in eGFR was 1.8 mL/min/1.73 m^2^ (95% CI: 1.9 to 1.7) in the S/V group, versus 2.4 mL/min/1.73 m^2^ (95% CI: 2.5 to 2.2) in the valsartan or enalapril group. This reno-protective effect of ARNI was consistent across the full spectrum of left ventricular ejection fractions (LVEFs), and the magnitude of benefit was similar across a broad range of baseline eGFR values, from 30 to 120 mL/min/1.73 m^2^.

McCausland and colleagues evaluated the PARAGON-HF trial and reported that the CV benefits—specifically reductions in HFH and CV death—were more pronounced in patients with lower baseline eGFR (eGFR ≤ 45 mL/min/1.73 m^2^) and lower LVEF ≤ 57% (hazard ratio: 0.66; 95% CI: 0.45–0.97) [55]. An editorial by Costanza raises the question of whether the CV benefits of S/V observed in HFpEF patients apply to those with severely reduced kidney function, as individuals with an eGFR < 30 mL/min/1.73 m^2^ at screening or <25 mL/min/1.73 m^2^ at randomization were excluded from trials. Furthermore, it remains unclear whether baseline eGFR modifies the therapeutic effects of ARNI. The author recognizes that a major barrier to the implementation of GDMT in clinical practice is the persistent fear of adverse kidney outcomes, despite consistent reassurance from large RTCs [56].

### 6.4. Sodium–Glucose Cotransporter-2 Inhibitors (SGLT2is)

The RASis have traditionally served as a foundational treatment for slowing the progression of diabetic kidney disease (DKD). However, the advent of SGLT2is marked a new era, significantly reshaping the management of not only DKD but also CKD and HF. In 2005, the first CV outcome trials (CVOTs) involving novel glucose-lowering agents such as SGLT2is were published [57,58,59]. The results of these trials led to a major paradigm shift in the management of T2DM. Since then, numerous additional CVOTs have been conducted, particularly in HF and chronic CKD, focusing on the effects of SGLT2is. These agents have consistently demonstrated cardiorenal benefits across various HF phenotypes, both in patients with and without diabetes. As a result, SGLT2is have become the foundational therapy in the management of HF.

All CVOTs involving SGLT2is have reported consistent reductions in MACE, CV death and HHF, although not all outcomes reached statistical significance. Nonetheless, the directional consistency of these benefits has been noteworthy. In 2021, McGuire and colleagues [60] conducted a meta-analysis of SGLT2i trials in T2DM, which confirmed a modest yet statistically significant reduction in MACE. Furthermore, SGLT2is were associated with a significant reduction in the risk of kidney disease progression, with consistent benefits observed across the drug class.

While the nephroprotective effects of SGLT2is were initially derived from secondary endpoints of CVOTs, direct confirmation came from dedicated kidney-focused trials. The Dapagliflozin and Prevention of Adverse Outcomes in Chronic Kidney Disease (DAPA-CKD) [61] trial and Canagliflozin and Renal Events in Diabetes with Established Nephropathy Clinical Evaluation (CREDENCE) trial [62] expanded the cardiorenal benefits of SGLT2is. The DAPA-CKD included patients with CKD (eGFR 25–75 mL/min/1.73 m^2^), with and without T2DM. Dapagliflozin significantly reduced the risk of a composite outcome that included a sustained eGFR decline of ≥50%, end-stage kidney disease or death from renal or CV causes. DAPA-CKD was the first trial in CKD patients with or without diabetes that demonstrated improved cardiorenal outcomes and reduced mortality, with a 39% relative risk reduction (HR, 0.61:CI 0.51–0.72) in the composite endpoint. The trial was stopped early due to overwhelming cardiorenal composite outcome efficacy, with consistent results across diabetic and non-diabetic populations. The CREDENCE trial reported a 30% relative risk reduction with canagliflozin for its primary kidney composite end point. Both the CREDENCE and DAPA-CKD trials enrolled high-risk patients meeting the KDI-GO staging criteria for very high risk (uACR > 300 and CKD stage 3B), demonstrating a kidney-protective effect. Similarly, the Study of Heart and Kidney Protection with Empagliflozin (EMPA-KIDNEY) [63] showed renoprotective effects across a broad range of patients at risk for CKD progression. Importantly, DAPA-CKD and EMPA-KIDNEY included participants with lower eGFRs (baseline eGFRs down to 20 mL/min/1.73 m^2^) and with wide variations in baseline albuminuria.

All trials involving SGLT2is showed a modest initial decline in eGFR, which is an expected effect following treatment initiation. This slight reduction has not been associated with adverse CV outcomes. In contrast, a decline in eGFR among placebo recipients was linked to worse CV outcomes. Notably, the acute drop in eGFR with SGLT2is is reversible, even with long-term use, and does not diminish the therapy’s cardiac or renal benefits [64]. The position paper from the European Society of Cardiology examines the effects of SGLT2is on kidney disease progression across various populations and reports consistent renal benefits throughout the spectrum [65]. Clinical trials have confirmed the safety and benefits of SGLT2is in patients with CKD, regardless of the presence of T2DM. Current guidelines recommend prioritizing their use in individuals at high risk for CV and renal complications.

### 6.5. Glucagon-like Peptide-1 Receptor Agonists (GLP-1RAs)

**Figure 4:**
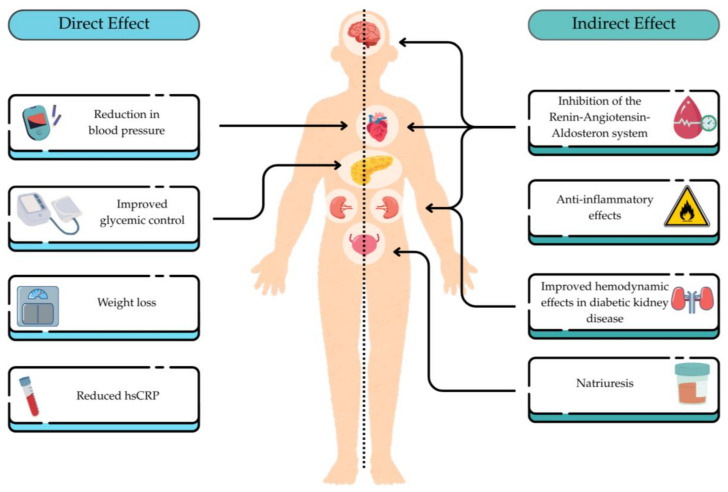
Proposed effects of GLP-1 receptor agonist. Adapted from reference [70]. Abbreviations: hsCRP = high-sensitivity C-reactive protein.

Despite many therapeutic advances for slowing the progression of DKD and CKD, a substantial number of patients remain at risk for progressive kidney function decline. There has been recent interest in GLP-1RAs due to emerging evidence suggesting potential renal benefits. GLP-1RAs have demonstrated kidney benefits, primarily through secondary outcomes in CVOTs. While most trials enrolled patients with early-stage CKD, three landmark studies—Liraglutide Effect and Action in Diabetes: Evaluation of Cardiovascular Outcome Results (LEADER) [66], Dulaglutide and Cardiovascular Outcomes in Type 2 Diabetes (REWIND) [67] and Semaglutide and Cardiovascular Outcomes in Patients with Type 2 Diabetes (SUSTAIN) [68]—highlighted the potential renoprotective effects of GLP-1RAs, particularly in composite kidney outcomes. Table 1 [69] shows a summary of landmark trials with GLP1-RAs highlighting both CV and renal outcomes. These benefits are hypothesized to result from both direct and indirect mechanisms [70], which are shown in Figure 4.

The Effects of Semaglutide on Chronic Kidney Disease in Patients with Type 2 Diabetes (FLOW trial) [71] is the first to specifically investigate the primary kidney-protective effects of a GLP-1RA in patients with eGFR as low as 25 mL/min/1.73 m^2^. Approximately 68% of individuals were classified as very high risk according to the KDIGO risk stratification categories based on eGFR and UACR. In this trial, semaglutide treatment led to a 24% relative risk reduction in the renal composite outcome compared to a placebo (HR: 0.76; 95%, CI: 0.66–0.88; *p* = 0.003). The composite endpoint included kidney disease progression (≥50% reduction in eGFR from baseline), initiation of dialysis or kidney transplantation and CV or kidney-related death. Notably, the number needed to treat (NNT for 3 years) was 20 to prevent one primary outcome event, 45 to prevent one MACE and 39 to prevent one all-cause death. The semaglutide group also experienced a 38% reduction in uACR and an average weight loss of 4.1 kg, as well as a lower incidence of serious adverse events compared to the placebo (49.6% vs. 53.8%). The mean annual decrease in the eGFR (total slope) was significantly lower in the semaglutide group by 1.16 mL per minute per 1.73 m^2^ (95% CI, 0.86 to 1.47; *p* < 0.001). The CV events, particularly HF, were fewer in the semaglutide group, with similar rates of AKI and severe hypoglycemia across both groups. The beneficial effects were consistent across different eGFRs and degrees of albuminuria.

The FLOW trial has some limitations. The low proportion of black patients (<5%) may restrict the generalizability of the findings to this population. Additionally, SGLT2i therapy was not mandated, as these agents were not the standard of care at the time of trial enrollment. Notably, more participants in the placebo group initiated SGLT2i therapy during the study therapy, likely in response to elevated albuminuria or hyperglycemia. This makes the observed benefits of semaglutide even more compelling, as the comparator group was increasingly receiving combined RASi/SGLT2i therapy. While there was no body max index (BMI) cutoff in the trial, the mean BMI was 31 kg/m^2^, raising the question of whether semaglutide would confer similar benefits in a non-obese population [71]. Nevertheless, the results of the FLOW trial will significantly influence clinical guidelines, positioning GLP-1RAs as the fourth pillar of CKD management in patients with T2DM.

The GLP-1RAs have also been explored in non-diabetic CKD, and results from the SELECT trial [72], which investigated the effects of semaglutide on heart disease and stroke in individuals who were overweight or had obesity, indicated that semaglutide may slow kidney function decline and reduce albuminuria in patients with non-diabetic CKD. To explore these findings further, researchers conducted a randomized controlled trial to assess the impact of once-weekly 2.4 mg subcutaneous semaglutide on albuminuria levels in patients with CKD, overweightness or obesity, but without diabetes [73]. After 24 weeks of treatment, semaglutide produced a clinically meaningful reduction in albuminuria in this population. These findings support the need for further studies on GLP-1 receptor agonists in non-diabetic CKD to broaden their potential therapeutic use.

## 7. From Evidence to Impact: Challenges and Opportunities of Implementing Best Practices in CKD Management

Optimal management of CKD involves addressing risk factors and implementing both nonpharmacological and pharmacological interventions to prevent disease progression and reduce complications.

### 7.1. Nonpharmacological Management of CKD and HF

Nonpharmacological approaches, including diet and physical activity, are vital in managing chronic kidney disease (CKD) and HF. Systematic reviews confirm that regular exercise improves mortality risk, cardiorespiratory fitness and quality of life (QoL) in all CKD stages, including dialysis and transplant patients. However, most CKD patients remain inactive. KDIGO recommends at least 150 min of moderate-intensity exercise weekly, tailored to individual capacity. Weight loss is encouraged in patients with obesity [28]. In HF, regular activity and structured cardiac rehab improve functional status and QoL [74].

KDIGO advises plant-based diets (e.g., (Dietary Approaches to Stop Hypertension (DASH), Mediterranean) and limiting animal-based and ultra-processed foods to slow CKD progression. Sodium intake should be <2 g/day to control blood pressure and proteinuria. In HF, recent evidence questions strict sodium and fluid restrictions; ESC now recommends salt < 5 g/day and fluid limits only in severe cases. The Fluid Restriction in Heart Failure versus Liberal Fluid Uptake (FRESH-UP) study suggests that liberal fluid intake is safe for patients with stable chronic HF and may even enhance well-being rather than a “one-size-fits-all” approach [75]. Every attempt should be made to optimize GDMT, which not only improves cardiac performance but also enhances renal hemodynamics.

Protein intake remains a topic of ongoing debate, with evolving guidelines. The 2024 KDIGO guidelines recommend a protein intake of 0.8 g/kg per day for patients with CKD Stages G3–G5. In contrast, the KDOQI guidelines from the National Kidney Foundation from US suggest a lower intake of 0.55–0.6 g/kg per day for non-diabetic patients with Stage 3 or 4 CKD. For those with diabetic kidney disease, a more moderate restriction of 0.6–0.8 g/kg per day is advised [76]. Reducing protein intake may help slow the decline in kidney function, delay CKD progression and reduce the risk of developing ESKD [77].

Experts suggest limiting potassium intake to less than 3.0 g/d in patients with eGFR < 30 mL/min per 1.73 m^2^ and even at earlier stages if hyperkalemia becomes a frequent finding [78]. Nevertheless, maintaining compliance with a low-potassium diet presents significant challenges, particularly for patients who must also limit sodium or carbohydrate intake because of CKD, HF or DM [79]. Moreover, the association between dietary potassium intake and serum potassium concentration remains poorly defined, as serum levels reflect potassium homeostasis over the preceding 24–48 h and may be influenced by acute events such as vomiting, diarrhea, hemorrhage or recent hospitalization [80]. Therefore, isolated serum potassium values may not accurately represent recent dietary intake.

The bioavailability of potassium varies depending on food sources. Processed foods tend to have higher potassium content due to additives [81,82], which are nearly 100% bioavailable. In contrast, plant-based, high-fiber foods generally have lower potassium absorption rates compared to low-fiber or processed alternatives. Furthermore, potassium absorption can be higher in diets lacking fiber. Animal-based protein sources are also associated with a greater risk of hyperkalemia compared to plant-based proteins, partly due to their higher potassium content and bioavailability [7]. Consequently, increased protein intake, particularly from animal sources, can exacerbate hyperkalemia risk.

Self-care is essential but often hindered by disease burden and complex regimens. Empowering patients through education and support improves outcomes, QoL and symptom control and reduces healthcare use. Referrals to renal or HF dietitians are strongly recommended.

### 7.2. Pharmacological Management of CKD and HF

Major risk factors for the development and progression of CKD—including HTN, dyslipidemia and DM—should be managed in accordance with established clinical guidelines. ACEi or ARBs are recommended to achieve a systolic blood pressure target of <120 mmHg. Statins may be beneficial for primary prevention in patients with early-stage CKD, particularly by reducing CV risk in high-risk individuals. However, initiating statin therapy in patients with dialysis-dependent CKD is generally not recommended. Nonetheless, continuing statins in those already receiving them prior to starting dialysis may be appropriate. In diabetes, tight glycemic control and agents like RASis, GLP-1RAs and SGLT-2is help reduce CKD progression.

**Figure 5:**
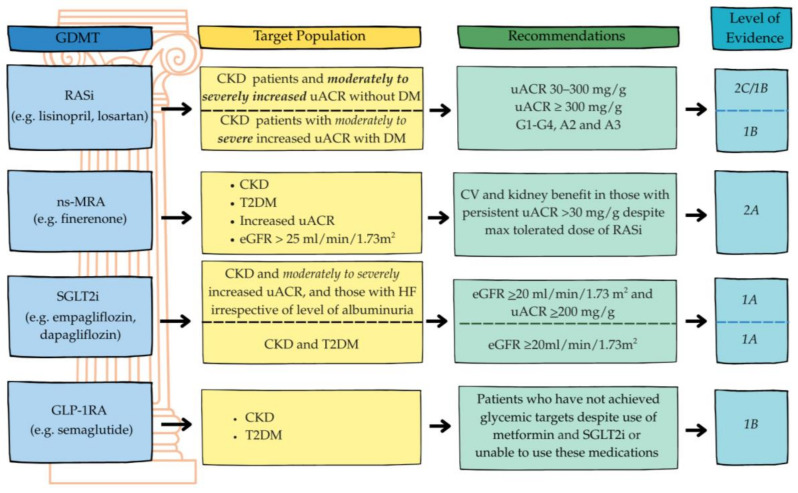
Four pillars of treatment for chronic kidney disease (CKD). Adapted Adapted with permission from reference [7]. Abbreviations: CKD = chronic kidney disease, CV = cardiovascular, eGFR = estimated glomerular filtration rate, GLP-1RA = glucagon-like peptide-1 receptor agonist, ns-MRA = nonsteroidal mineralocorticoid receptor antagonist, RASi = renin-angiotensin system inhibitor, SGLT2i = sodium–glucose co-transporter 2, T2DM = Type 2 diabetes mellitus, uACR = urine albumin-to-creatinine ratio.

Pharmacologically, all patients should receive four pillars of treatment for cardiorenal protection with CKD and T2DM, specifically maximally tolerated doses of RASis—either ACEis or ARBs—as first-line therapy. Figure 5 summarizes the indications for the four pillars of CKD management with or without DM. Beyond RASis, other agents should be considered based on albuminuria severity and comorbid conditions [7].

In clinical practice, it is common to see the rapid sequencing of medications, particularly in the management of chronic conditions such as myocardial infarction, HFrEF and respiratory diseases. The Safety, Tolerability and Efficacy of Rapid Optimization, Helped by N-Terminal Pro–Brain Natriuretic Peptide Testing of Heart Failure Therapies (The STRONG-HF) trial demonstrated that after ADHF, all efforts should be made to rapidly up titrate to optimal doses of the three and (likely) four pillars of HF medications, combined with close follow-up. This approach significantly reduced the risk of death or HF readmission [83]. A post hoc analysis revealed that patients who received higher GDMT doses and had their medications up titrated within two weeks experienced lower rates of HF readmission or death over six months, along with improved QOL. Conversely, patients with lower blood pressure, greater congestion or reduced kidney function (eGFR < 30 mL/min/1.73 m^2^) received lower GDMT doses. Frequent, protocol-guided reassessment and adjustment of therapy based on safety indicators likely contributed to the treatment’s success [84]. Notably, over half of the patients met at least one safety trigger during follow-up, yet their 180-day outcomes were comparable to those who did not—further supporting the overall benefit of rapid GDMT intensification over usual care [85]. The STRONG-HF trial demonstrates the safety and value of GDMT titration in patients with HFrEF; however, in clinical practice, treatment must be individualized to account for comorbidities. Since patients with CKD were not integrated into STRONG-HF, management in this population requires a tailored approach with close monitoring of renal function.

Additionally, appropriate sequencing of GDMT can enhance the safety and tolerability of certain medications—for instance, the risk of hyperkalemia associated with MRAs can be reduced by adding SGLT2is. Rapid initiation and titration of HF foundational therapies within four weeks have also been associated with significant reductions in mortality and hospitalizations [86]. Multiple clinical trials have been recently published to evaluate the simultaneous initiation of combination of two therapies in patients with CKD and T2DM to reduce CV and kidney-related risks leading to improvement in CV and kidney free events [87,88,89,90].

Although each therapy individually reduces CV and kidney events, evidence indicates that significant residual risks persist. Therefore, in some individuals, simultaneous use of therapies may offer additional risk reduction [91]. However, implementing this strategy in real-world clinical practice presents notable challenges. From the provider’s standpoint, several barriers can hinder the implementation of multiple therapies simultaneously. These include knowledge gaps and limited familiarity with current treatment guidelines. Reliance on subspecialists to initiate certain therapies can disproportionately disadvantage patients who have limited access to specialty care, further exacerbating health disparities [92]. Patient-specific factors such as tolerability and introduction of multiple new medications can lead to non-adherence. Many essential therapies are often stopped during episodes of AKI and may not be restarted even after kidney function recovers. This practice can be counterproductive, as it may reduce the long-term benefits of these medications. In a study with CKD, T2DM and a recent AKI event, the use of SGLT2is was linked to a 28% reduction in the risk of CKD progression and a 25% lower risk of recurrent AKI [93]. Despite renal benefits, around one in ten individuals with CKD stops taking SGLT2is within six months, most often due to a reduction in eGFR, which accounts for nearly two-thirds of the discontinuations [94]. Additionally, the cost of starting four new medications at once can be prohibitive, particularly for patients with limited insurance coverage. Out-of-pocket expenses may further worsen disparities in care, especially among underserved populations. Although combination therapies are widely promoted for various chronic diseases, the challenge in cardiorenal care remains identifying the most effective, scalable and patient-centered treatment regimens. There is no clear validated algorithm to guide parallel or rapid sequential initiation of therapies in CKD.

## 8. Conclusions

In conclusion, a comprehensive and holistic approach is essential to address the progression of CKD. The aim of therapy is to slow the permanent loss of functional nephrons, thereby maintaining GFR and postponing the development of kidney failure. Early diagnosis using baseline laboratory data and addressing modifiable risk factors (DM, HTN, obesity) are key to preventing CV events. Initial screening should include both eGFR and uACR, followed by repeat testing at intervals ranging from annually to every 3–4 months, depending on the clinical context and individual risk stratification. Initiation of GDMT should not be delayed, and treatment should be individualized based on patient-specific factors. All four pillars of GDMT should be optimized for both CKD and HF. Therapies should be reinitiated as soon as kidney function improves to achieve long-term benefit. Managing patients with multiple chronic conditions, especially the intersecting challenges of kidney and CV diseases, demands a nuanced balancing of competing therapeutic priorities. Clinicians must weigh each medication’s indication, any secondary benefits and the practical hurdles of treating several illnesses at once. Additionally, patient education should emphasize lifestyle modifications and strategies to enhance medication adherence.

## Figures and Tables

**Table 1:** GLP1-RA cardiovascular and renal outcomes. Direct comparisons not possible due to differing study designs. Adapted from reference [69] * *p* < 0.05.

Trial	Agent (Dose)	Median Follow-Up (yrs)	Composite Cardiovascular Endpoint	Exploratory Renal Composite Endpoint	Macroalbuminuria	Worsening eGFR	Renal Replacement Therapy
LEADER	Liraglutide 1.8 mg/d	3.8	13% relative risk reduction HR: 0.87 (95% CI: 0.78–0.97) *p* = 0.01	0.78 [0.67–0.92] *	0.74 [0.60–0.91] *	0.89 [0.67–1.19]	0.87 [0.61–1.24]
REWIND	Dulaglutide 1.5 mg/wk	5.4	11% relative risk reduction HR: 0.89 (95% CI: 0.79–0.99) *p* = 0.026	0.85 [0.77–0.93] *	0.77 [0.68–0.87] *	0.89 [0.78–1.01]	0.75 [0.39–1.44]
SUSTAIN-6	Semaglutide 0.5/1 mg/wk	2.1	26% relative risk reduction HR: 0.74 (95% CI: 0.58–0.95) *p* =< 0.001	0.64 [0.46–0.88] *	0.54 [0.37–0.77] *	1.28 [0.64–2.58]	0.91 [0.40–2.07]

## Data Availability

Not applicable.

## Abbreviations

The following abbreviations are used in this manuscript:

ACEi	Angiotensin-converting enzyme inhibitors
ADHF	Acute decompensated heart failure
AKI	Acute kidney injury
ARB	Angiotensin II receptor blockers
ARNI	Angiotensin receptor-neprilysin inhibitor
CKD	Chronic kidney disease
CV	Cardiovascular
CVOTs	CV outcome trials
DM	Diabetes
eGFRcr	Estimated GFR based on serum creatinine
eGFRcr-cys	Cystatin C–based eGFR
ESKD	End-stage kidney disease
GDMT	Guideline directed medical therapy
GFR	GFR glomerular filtration rate
GLP-1RA	Glucagon-like peptide-1 receptor agonists
HFH	Heart failure hospitalization
HFmrEF	Heart failure with mildly reduced ejection fraction
HFpEF	Heart failure with preserved ejection fraction
HFrEF	Heart failure with reduced ejection fraction
HTN	Hypertension
KRT	Kidney replacement therapy
MACE	Major adverse CV events
RASi	Renin-angiotensin system inhibitors
RAAS	Renin-angiotensin-aldosterone system
SCr	Serum creatinine
SGLT2i	Sodium–glucose cotransporter 2 inhibitors
S/V	Sacubitril/valsartan
T2DM	Type 2 diabetes mellitus
uACR	Urine albumin-to-creatinine ratio
WRF	Worsening renal function
